# Academic Primer Series: Key Papers About Competency-Based Medical Education

**DOI:** 10.5811/westjem.2017.3.33409

**Published:** 2017-05-01

**Authors:** Robert Cooney, Teresa M. Chan, Michael Gottlieb, Michael Abraham, Sylvia Alden, Jillian Mongelluzzo, Michael Pasirstein, Jonathan Sherbino

**Affiliations:** * Geisinger Medical Center, Department of Emergency Medicine, Danville, Pennsylvania; † McMaster University, Department of Medicine, Division of Emergency Medicine, Hamilton, Ontario, Canada; ‡ Rush University Medical Center, Department of Emergency Medicine, Chicago, Illinois; § University of Maryland School of Medicine, Department of Emergency Medicine, Baltimore, Maryland; ¶ University of New Mexico, Department of Emergency Medicine, Albuquerque, New Mexico; || University of California San Francisco, Department of Emergency Medicine, San Francisco, California; # Drexel University College of Medicine, Department of Emergency Medicine, Philadelphia, Pennsylvania

## Abstract

**Introduction:**

Competency-based medical education (CBME) presents a paradigm shift in medical training. This outcome-based education movement has triggered substantive changes across the globe. Since this transition is only beginning, many faculty members may not have experience with CBME nor a solid foundation in the grounding literature. We identify and summarize key papers to help faculty members learn more about CBME.

**Methods:**

Based on the online discussions of the 2016–2017 ALiEM Faculty Incubator program, a series of papers on the topic of CBME was developed. Augmenting this list with suggestions by a guest expert and by an open call on Twitter for other important papers, we were able to generate a list of 21 papers in total. Subsequently, we used a modified Delphi study methodology to narrow the list to key papers that describe the importance and significance for educators interested in learning about CBME. To determine the most impactful papers, the mixed junior and senior faculty authorship group used three-round voting methodology based upon the Delphi method.

**Results:**

Summaries of the five most highly rated papers on the topic of CBME, as determined by this modified Delphi approach, are presented in this paper. Major themes include a definition of core CBME themes, CBME principles to consider in the design of curricula, a history of the development of the CBME movement, and a rationale for changes to accreditation with CBME. The application of the study findings to junior faculty and faculty developers is discussed.

**Conclusion:**

We present five key papers on CBME that junior faculty members and faculty experts identified as essential to faculty development. These papers are a mix of foundational and explanatory papers that may provide a basis from which junior faculty members may build upon as they help to implement CBME programs.

## INTRODUCTION

While competency-based medical education (CBME) can trace its roots to the early 1970s, it has only been in the last 15 years that the concept has become mainstream within medical education. 1 This adoption likely stems from the combined influence of changing regulatory requirements, global interest in the adoption of competency frameworks, public demand for higher quality care, and increased physician and health system accountability. 2 Providing higher quality care and reducing practice variation are significant driving factors for the adoption of CBME as multiple studies demonstrate systemic failures to improve care 3 and evidence that residency training drives future performance. 4–6

CBME has become a global phenomenon. In the United States, the Accreditation Council on Graduate Medical Education (ACGME) introduced six domains of clinical competence (patient care, medical knowledge, practice-based learning and improvement, interpersonal and communication skills, professionalism, systems-based practice) in 1998. 7 In 2013, these original competencies were further refined through the Next Accreditation System and the creation of milestones within residency programs. 8 Similarly, Canada introduced the CanMEDS Framework that defines seven roles (medical expert, communicator, collaborator, manager, health advocate, scholar, and professional). 1 This framework is used in more than 58 jurisdictions in dozens of countries in five continents. 9 Additional frameworks exist in Australia 10 and Europe, including the United Kingdom (Tomorrow’s Doctor), 11 and Scotland (the Scottish Doctor). 12

The current status of residency education can be described as a structure- and process-based system. Within this model, trainees are exposed to learning content for a specific amount of time. 13 Assessment within the system focuses predominantly on knowledge acquisition. Application of knowledge, skills, and attitudes are rarely assessed within the traditional system, leading to inadequate demonstration of preparation for independent practice. 14 Adoption of CBME seeks to correct the shortcomings that exist within the current system. Principles of CBME include a shift toward the use of defined competencies required for practice, staged progression of increasing responsibility/independence, tailored learning, and programmatic assessment. 15 Early-career clinician educators will be expected to navigate the challenges currently facing healthcare while being called upon to translate these concepts into workable solutions that meet the needs of the profession and society

The Academic Life in Emergency Medicine (ALiEM) Faculty Incubator was created partly to give early-career educators a solid exposure to topics that are relevant to the 21st century medical educator. During our yearlong Faculty Development Incubator, CBME was the focus of one module. This paper is a synthetic, narrative review highlighting important literature that may assist junior educators who are seeking to learn more about the design and theoretical foundation of CBME.

## METHODS

From August 1–31, 2016, the ALiEM Faculty Incubator discussed CBME. The online discussions involved both junior faculty participants and faculty mentors. As online discussions organically explored the topic of CBME, the titles of papers that were cited, shared, and discussed within the online discussion forum were curated.

This list of manuscripts was augmented with the following: 1) a Google Hangout On Air (GHOA) with Dr. Stan Hamstra of the ACGME, and 2) a call for important CBME papers on Twitter. We requested participants of the #FOAMed and #MedEd online communities to nominate other important CBME papers.

The authorship team then conducted a four-round voting process, inspired by the Delphi methodology similar to previous papers that covered educational scholarship, 16 team collaboration, 17 educational theory, 18 and educational consults. 19 This was not a traditional Delphi methodology since our selection panel comprised both novices (i.e. junior faculty members, participants in the Faculty Incubator) and experts in the field (i.e., experienced clinician educators, all of whom have published greater than 10 peer-reviewed publications, who serve as mentors and facilitators of the ALiEM Faculty Incubator). However, we intentionally sought to involve both junior and experienced clinician educators to ensure we selected papers that would be of use to a spectrum of educators. The first round asked the raters to use a seven-point scale to rate the relevance of the paper for our intended target audience. The second round asked them to recommend whether the manuscript might be worthwhile for which a summary would be written. The third round asked them to further refine the list more restrictively, only allowing our selection panel to vote for five papers. Due to a tie among three candidate papers in the third round, a fourth round of voting then was completed to determine which of these three papers would be included in our top five papers.

## RESULTS

Our initial review of the ALiEM Faculty Incubator discussion on CBME thread yielded a total of five articles, which were mentioned by mentors and the junior Faculty Incubator participants. The expert GHOA discussion added another eight papers, and the social media calls yielded an additional 10 articles. There were two duplicates. The three-round voting procedure allowed our team to generate a rank-order listing of all these papers in order of relevance, from the most important to the least important. The citations and our ratings of the remaining 21 papers are listed in our Table.

## DISCUSSION

Presented here is a summary and commentary of the top papers.

### 1. Frank JR, Snell LS, Cate OT, et al. Competency-based medical education: theory to practice. *Med Teach*. 2010;32(8):638–45.^15^

#### Summary

This paper is best described as “proceedings” from an international conference convened to explore the emerging concepts of CBME. The specific aims were to review current literature, identify controversies, propose standard definitions, and identify future directions for academic exploration. The sections are broken down into the rationale for CBME, which delves into the principles that support CBME. The article contrasts the differences with traditional medical education, where CBME focuses on abilities, outcomes, learner-centeredness, and de-emphasizes time-based training. The second section focuses on definitions that are useful in CBME. The authors define competence as, “possessing the required abilities in all domains in a certain context at a defined stage of medical education or practice” (p 641). 15 They also distinguish between *dyscompetence*, which denotes that the learner is only partially unable to meet the goals, and *incompetence*, which implies that the learner is deficient in *all* areas of the skill or ability. The final section is a discussion of both advantages and hurdles that are to be expected with the implementation of CBME.

#### Relevance to Junior Faculty Members

This article is pertinent to junior faculty. It provides a background, explaining the societal and education influences of the CBME movement The International CBME Collaborators do stellar work in focusing the reader on the rationale for a CBME curriculum. This article’s table is filled with many pearls that translate the principles of CBME to the practical elements of a curriculum. The definitions provided are also important to help eliminate confusion and ensure a common lexicon when discussing CBME. Probably the most useful section in the article is the final section on the perils and promise of CBME. The main drawback to the implementation of CBME seems to be that the resources required.

#### Considerations for Faculty Developers

Faculty developers should use this foundational paper to orient junior faculty to the key definitions relevant to CBME. The paper also provides an effective contrast between traditional medical education curricula and CBME. With its thorough but readable review of the literature that informs the origins of the CBME movement, this manuscript would be an excellent choice as pre-reading (i.e., background) material for any faculty development course seeking to introduce junior faculty to the principles of CBME.

### 2. Carraccio C, Englander R, Van Melle E, et al. Advancing Competency-Based Medical Education: A Charter for Clinician-Educators. *Acad Med.* 2016;91(5):645–9. 20

#### Summary

This paper presents a charter, developed by the ICBME Collaborators, with the goal of establishing a conceptual model to be used when discussing, developing and implementing CBME. There is burgeoning international support for adoption of CBME in medical education. Although there is little formal evidence supporting this model, advocates cite it as the product of sound education theory and note the shortfalls of the current system of medical education. It is important to understand that there are major barriers to adoption, including logistical concerns, and the implementation process and outcomes must be carefully and transparently evaluated. The foundation of CBME entails a focus on outcome abilities, defined by patient and societal needs, and a de-emphasis on time-based training. 21 The charter then lays out 13 fundamental principles that are the foundation of CBME implementation.

The principles can be broadly categorized into themes. First is a refocusing of the relationship between medical providers and the populations they serve. The education of future medical providers should be determined by the needs of the populations they will serve, and there must be transparency for all stakeholders both within medical education and surrounding outcomes. Secondly, the role of the learner needs to be redefined. They must be empowered to take control of their education. As the primary focus of education and training shifts to desired outcomes for learners, effective and efficient assessment is key to timely and appropriate progression of learners through their education. These transitions will be based on achievement of competence rather than time. Moreover, the traditional stages of medical education should be supplanted with a more seamless educational trajectory that extends throughout one’s career. Thirdly, commitment from medical educators is imperative. They are responsible for teaching, assessing and role modeling the competencies that learners are being taught, and they must be provided with faculty development to keep them up to date on these competencies. They are also responsible for balancing patient safety with teaching and learner development. Finally, as CBME is implemented it must be studied and shared. Assessment of programs will provide feedback as to the effectiveness of training programs and direct future educational innovations. Additionally, open sharing of educational programs locally, internationally and among interprofessional training programs will allow for high-quality training programs while minimizing the resource-intensive nature of educational innovation.

#### Relevance to Junior Faculty Members

Governing bodies within medical education are transitioning from training organized by time to outcome-based training. The content, structure, and functionality of training programs will continue to change as the definition of a competent physician is explored and defined, and learners are expected to achieve a wider set of abilities. It is important to understand what CBME is, what it looks like in its idealized form, and the principles that it is built on. It is through this lens that frameworks for assessing learner performance such as competencies, milestones and entrustable professional activities (EPA) make sense. This shift in what is defined as success in training will require innovative curricula and new methods of evaluation. As junior faculty are often recruited for assistance in correcting perceived deficits within a program, a good understanding of CBME will facilitate creation of high-quality educational products. Conversely, looking at a training program through the filter of CBME principles may highlight areas of possible improvement and guide junior faculty into areas of personal interest. Finally, just as the study of medicine builds on a solid foundation of human anatomy, medical education should build on a solid foundation of medical education theory.

#### Considerations for Faculty Developers

For faculty developers this paper provides guidelines to consider when developing new (or modifying existing) curricula using a competency-based design. Principles such as “serving the health needs of a population,” “commitment to transparency” and “balancing learner needs with patient safety” among others have significant influence on how a curriculum is designed and operates. This paper argues for organizing principles of CBME that faculty developers must consider in their curricular innovations. The argument concludes that the adoption of these principles will lead to a robust and effective curriculum.

### 3. Carraccio C, Englander R, Gilhooly J, et al. Building a Framework of Entrustable Professional Activities, Supported by Competencies and Milestones, to Bridge the Educational Continuum. *Acad Med.* 2017;92(3):324–30. 22

#### Summary

This paper provides an introduction to two main features of CBME: entrustable professional activities and milestones. EPAs are sentinel tasks (i.e., work) tailored to a specific discipline (i.e., specialty) and performed in an authentic environment (e.g., the emergency department [ED], not in a simulated fashion). Typically, EPAs contain multiple competencies from multiple domains. This unique assessment tool uses a scale of entrustment (i.e., progression from close to indirect supervision to independence) to assess the competence of a trainee. 23–25 As learners’ progress towards more complex stages of training, their performance on multiple EPAs that sample multiple domains of competence help to determine the level of supervision required. Separate from EPAs, milestones describe specific performance at a specific stage of training relevant to a specific competency. Much smaller than an EPA, and not necessarily a clinical task, milestones provide a marker of progression, providing guidance to both trainees and faculty about progression towards global attainment of competence (i.e. readiness for unsupervised practice). For example, the ability to recognize and care for a critically ill patient in the ED would represent an EPA. A milestone would consist of progressively increasing levels of sophisticated management, beginning with the recognition of abnormal vitals signs and progressing to development of a protocol to improve the management or transfer of a critically ill patient. 26 The authors document the overlapping features between EPAs and milestones and their approach to integrating the two components.

#### Relevance to Junior Faculty Members

Through a discussion of EPAs, this paper emphasizes the importance of assessing learners throughout various experiences with an integrated pathway. Learners progress along individual trajectories of increasing competence and independent practice for specific sentinel abilities. For example, an undergraduate medical student is expected to be a secondary participant in resuscitation, while a junior resident will perform key critical procedures and the senior resident will lead the entire team. Thus, one must be cognizant of matching performance on an EPA to a specific stage of training. Similarly, EPAs are typically content specific, meaning performance of one EPA does not predict performance on another. How a learner performs on a spectrum of EPAs (excelling, or requiring further attention) allows a faculty member to co-produce with the learner a tailored learning plan moving forward. As an example, if a learner is able to perform a central line insertion without direct supervision, but is struggling with communicating with consultants, the learner should be given increasing independence with the former, while providing closer monitoring and feedback for the latter, so as to maximize both the learner’s time and instructor’s teaching efforts.

#### Considerations for Faculty Developers

The implementation of the Next Accreditation System in the United States introduced the concept of educational milestones – measurable markers of progression. When combined with EPAs all of the competencies (milestones) and work (EPA) of a specialty can be assessed using a systematic process that emphasizes authentic performance. Changes to assessment will be the most obvious innovation or change in a new CBME model. Unfortunately, many frontline teaching faculty may not be prepared for the implementation of CBME and EPAs. 27 Early success will require significant faculty development in assessment. Faculty developers will find this article useful in illustrating the alignment between the milestones and EPAs. Furthermore, this article provides a feasible example of how to apply EPAs across the trajectory from medical school to practice. Providing this example to faculty members will help to promote understanding of how competencies, milestones, and EPAs align within a well-designed assessment system.

### 4. Carraccio C, Wolfsthal SD, Englander R, et al. Shifting paradigms: from Flexner to competencies. *Acad Med*. 2002;77(5):361–7. 13

#### Summary

This paper reviews the literature on CBME as it stood in the early 21st century. CBME was first introduced in the medical literature in the 1970s. The forces behind this paradigm shift from structure and process-based to competency-based paradigms was driven by the cultural climate of the 1960s and 1970s. Advocacy for this shift was seen in a variety of professions and education levels. Pressures from the public, public health leaders, and professional organizations for increasing accountability helped push this paradigm change. The movement started with emphasizing the differences between what the current models were (structure- and processed-based paradigms) and the ideals of competency-based educational programs. During the 1970s, the medical literature focused on defining the competencies and less on determining competency components, evaluation of the competencies, and the overall assessment of the process. The authors suggest that lack of assessment strategies may have led to delays in implementing a full competency-based curriculum in medical education.

It was not until the turn of the century that CBME implementation in the health professions became a reality. Initiatives by various institutions focused on engaging faculty, administration, and learners in adopting competency-based education. The authors encourage more medical education research to support the outcomes of CBME.

#### Relevance to Junior Faculty Members

This article gives a historical perspective on the actual definition and implementation of CBME in medical education. Key differences between structure- and process-based education programs and competency-based programs (Table 1, page 362) 13 are described. The paper provides insight as to why there was a lag between widespread implementation of CBME from its development in the 1970s. Defining the four steps of CBME curriculum design is a critical insight for junior faculty members. The lessons from the late 20th century highlight the importance of faculty, learner, administrative, and key stakeholder engagement to create change in medical education.

#### Considerations for Faculty Developers

This article represents one of the earliest reviews of the transition to CBME within the U.S. medical education system. While competency constructs have been further refined since the publication of this article, educators will find it helpful to review the methodology for identifying and describing competence and how it informs curriculum design (page 363). 13 Understanding the difference between the CBME framework and the current system can be difficult. This article provides an often-cited table (Table 1, page 362) 13 that illustrates the differences between the historical structure- and process-based system and the emerging competency-based system.

### 5. Nasca TJ, Philibert I, Brigham T, et al. The next GME accreditation system—rationale and benefits. *N Engl J Med*. 2012;366(11):1051–6. 8

#### Summary

The Accreditation Council for Graduate Medical Education (ACGME) initially accredited graduate medical education residency programs on multiple factors, including program structure, quality of formal teaching, service to education balance, resident and faculty feedback, and financial benefits to residents. The ACGME developed the Next Accreditation System (NAS), which has been fully implemented since 2014. This new system prioritizes education outcomes as a significant determinant in residency program accreditation. With the inception of the NAS, discipline-specific milestones are used to assess trainees in the categories of patient care, medical knowledge, practice-based learning and improvement, interpersonal and communication skills, professionalism, and systems-based practice. Furthermore, on-site reviews every four to five years informed by templated program information forms have been replaced by annual data collection informed by self-critique and backed by a 10-year site visit. With the changes in the accreditation process, individual programs are allowed to innovate. Finally, the NAS allows disciplines to change the milestones as stakeholders’ expectations of the specialty change with time.

#### Relevance to Junior Faculty Members

With the full implementation of the NAS, junior faculty have specific milestones to anchor their assessments of trainees, and education innovation is encouraged by the ACGME. Junior faculty will benefit by understanding the framework of the previous accreditation system and what the vision for the future entails with the implementation of the NAS.

#### Considerations for Faculty Developers

This is a now-classic article about the changes that the ACGME underwent in the first two decades of the new millennia. This paper outlines the ACGME milestones project and the rationale for the change, making manifest the abstract nature of competencies. To complement this paper, it is important to draw linkages between the thoughts displayed in this paper and the outcomes-based education (OBE) movement that occurred in the 1970s. 28 Of note, within general education (and certainly elementary and secondary education), OBE has been a controversial subject. 28,29 Reading education literature on the pitfalls elementary and secondary school teachers have encountered may provide faculty developers with a new lens through which to view their own implementation and design challenges with CBME. A good primer on OBE from the medical education literature is the five-part AMEE Guide series (No. 14) 30–34

## HONORABLE MENTION

### ten Cate O, Hart D, Ankel F, et al. Entrustment Decision Making in Clinical Training. *Acad Med*. 2016;91(2):191–8. 35

While not in the top five papers, this paper discusses an important principle of CBME – entrustment (i.e., the delegation of responsibility to a trainee to complete a task via indirect supervision). Entrustable professional activities are a new education concept that are highly influential in CBME assessment. This paper provides 1) a definition of entrustment, 2) a description of entrustment (supervision) levels and the trainee-supervisor dyad, 3) factors involved in entrustment decisions, and 4) a process for using grounding summative assessments in an entrustment model.

## LIMITATIONS

As with our previous papers, we did not design this study to be an exhaustive, systematic search of the literature. We used expert consultation and an open social media call via Twitter using hashtags #MedEd & #FOAMed to expand our search. Given this approach, it is possible that we introduced an availability bias into our sample, though this is unlikely given the breadth of the submissions considered. In addition, we did not restrict submissions from alternative publications or the grey literature. As with prior publications within this series, we aimed to provide a succinct review of high-yield papers for faculty members to use as a starting point to explore the important concepts within CBME. Finally, we make no claims that this is a definitive list of all the papers that are the exclusive body of literature all educators should know, but rather we feel that these are five papers that we have determined via the process described to isolate some readings that novice educators and those teaching them may find most useful. We feel that we may have selected a valid grouping of papers, since the majority of our top five papers are highly cited papers with a cumulative total of more than 500 citations.

## CONCLUSION

We provide a reading list on the topic of CBME that may serve as a primer for junior faculty members engaged in medical education. Faculty developers will find this list useful as a foundational series of articles addressing the history of the development of the CBME movement, defining themes within CBME, important principles to consider in the design of curricula, and a rationale for changes to accreditation that are inevitable with the adoption of CBME.

## Figures and Tables

**Table:** The complete list of educational scholarship literature related to competency-based medical education that was collected by the authorship team.

Citation	Round 1 initial mean scores (SD) max score 7	Round 2 % of raters that endorsed this paper	Round 3 % of raters that endorsed paper in this round	Round 4 tie break round	Top 5 papers
Frank JR, Snell LS, Cate OT, et al. Competency-based medical education: theory to practice*. Med Teach*. 2010;32(8):638–45.	6.5 (0.76)	100%	100%		1
Carraccio C, Englander R, Van Melle E, et al. Advancing Competency-Based Medical Education: A Charter for Clinician-Educators. *Acad Med*. 2016;91(5):645–9.	6.4 (0.74)	100%	100%		2
Carraccio C, Englander R, Gilhooly J, et al. Building a Framework of Entrustable Professional Activities, Supported by Competencies and Milestones, to Bridge the Educational Continuum. *Acad Med*. 2017;92(3):324–330.	6.1 (0.83)	100%	87.5%		3
Carraccio C, Wolfsthal SD, Englander R, et al. Shifting paradigms: from Flexner to competencies. *Acad Med*. 2002;77(5):361–7.	5.6 (0.92)	87.5%	50%		4
Nasca TJ, Philibert I, Brigham T, et al. The next GME accreditation system—rationale and benefits. *New England Journal of Medicine*. 2012 Mar 15;366(11):1051–6.	5.4 (1.19)	75%	37.5%	62.5%	5
ten Cate O, Hart D, Ankel F, et al. Entrustment decision making in clinical training. *Acad Med*. 2016 Feb;91(2):191–8.	5.8 (1.23)	62.%	37.5%	37.5%	Honorable Mention
Chan T, Sherbino J; McMAP Collaborators. The McMaster Modular Assessment Program (McMAP): a theoretically grounded work-based assessment system for an emergency medicine residency program. *Acad Med*. 2015;90(7):900–5.	5.6 (1.06)	75%	37.5%	0%	
Hodges BD. A tea-steeping or i-Doc model for medical education? *Acad Med*. 2010 Sep;85(9 Suppl):S34–44.	5.6 (1.51)	50%	0%		
ten Cate O, Scheele F. Competency-based postgraduate training: can we bridge the gap between theory and clinical practice? *Acad Med*. 2007;82(6):542–7.	5.6 (1.19)	75%	25%		
Konopasek L, Norcini J, Krupat E. Focusing on the Formative: Building and Assessment System aimed at student growth and development. *Acad Med*. 2016 Mar 29. [Epub ahead of print]	5.5 (1.07)	25%	0%		
Holmboe ES, Ward DS, Reznick RK, et al. Faculty development in assessment: the missing link in competency-based medical education. A*cad Med.* 2011;86(4):460–7.	5.4 (1.51)	50%	12.5%		
Asch DA, Nicholson S, Srinivas SK, et al. How do you deliver a good obstetrician? Outcome-based evaluation of medical education. *Acad Med*. 2014;89(1):24–6.	4.75 (1.49)	0%	0%		
Gofton WT, Dudek NL, Wood TJ, et al. The Ottawa surgical competency operating room evaluation (O-SCORE): a tool to assess surgical competence. *Acad Med*. 2012;87(10):1401–7.	4.75 (0.89)	0%	0%		
Batalden P, Leach D, Swing S, et al. General competencies and accreditation in graduate medical education. *Health Aff* (Millwood). 2002;21(5):103–11.	4.6 (1.19)	37.5%	12.5%		
McGaghie WC, Miller GE, Sajid AW, Telder TV. Competency-based curriculum development on medical education: an introduction. *Public Health Pap*. 1978;(68):11–91.	4.6 (1.69)	37.%	0%		
Gingerich A, Regehr G, Eva KW. Rater-based assessments as social judgments: Rethinking the etiology of rater errors. *Acad Med*. 2011;86(10):S1–7.	4.4 (0.92)	0%	0%		
Chen C, Petterson S, Phillips R, et al. Spending patterns in region of residency training and subsequent expenditures for care provided by practicing physicians for Medicare beneficiaries. *JAMA*. 2014;312(22):2385–93.	3.9 (1.89)	0%	0%		
Cook DA, Brydges R, Ginsburg S, et al. A contemporary approach to validity arguments: a practical guide to Kane’s framework. *Med Educ*. 2015;49(6):560–75.	3.8 (1.67)	0%	0%		
Landrigan CP, Parry GJ, Bones CB, et al. Temporal trends in rates of patient harm resulting from medical care. *New Eng J Med*. 2010;363(22):2124–34.	3.8 (1.98)	0%	0%		
Messick S. Validity of psychological aassessment. *American Psychologist*. 1995;50(9):741–9.	3.5 (1.20)	0%	0%		
Jay A. How to run a meeting. *Harv Bus Rev*. 1976;54:1–12.	3.3 (2.43)	0%	0%		
